# Reversal of Acute Liver Injury Post–Roux-en-Y Gastric Bypass With Total Parenteral Nutrition

**DOI:** 10.14309/crj.0000000000001991

**Published:** 2026-02-05

**Authors:** Carla Daou, Fatma Mahmoud, Doa'a Alkhader, Abdullah Shatnawei, Sulieman Abdal Raheem

**Affiliations:** 1 Digestive Disease Institute, Cleveland Clinic Abu Dhabi, Abu Dhabi, UAE

**Keywords:** total parenteral nutrition, acute liver injury, Roux-en-Y gastric bypass, liver transplant

## Abstract

Acute liver injury is a rare yet serious complication of bariatric surgery, particularly Roux-en-Y gastric bypass. We report the case of a 39-year-old woman with a history of hepatitis A and metabolic dysfunction–associated steatotic liver disease who developed acute liver injury after significant weight loss and malnutrition post–Roux-en-Y gastric bypass. On admission, she presented with steatorrhea, jaundice, and severe hepatic dysfunction. Investigations revealed vitamin A and D deficiencies, steatosis, fibrosis (F3), and severe malabsorption. Total parenteral nutrition was initiated 3 days postadmission and continued for 5.5 months. The patient achieved 8kg weight gain, normalization of liver function tests and vitamin levels, and reversal of steatosis and fibrosis (F0/S2 on repeat Fibroscan). Clinically, her symptoms resolved, and she no longer required a liver transplant. This case highlights the potential of early, sustained total parenteral nutrition in reversing acute liver injury secondary to malnutrition and underscores the importance of comprehensive nutritional support postbariatric surgery.

## INTRODUCTION

Bariatric surgery is widely used for obesity and related comorbidities including metabolic dysfunction–associated steatohepatitis and metabolic dysfunction–associated steatotic liver disease (MASLD).^1^ Roux-en-Y gastric bypass (RYGB) improves metabolic profiles and reduces hepatic steatosis in patients with obesity and MASLD. Paradoxically, severe hepatic complications such as steatohepatitis and acute liver injury have been reported post-operatively, especially with rapid weight loss, protein-calorie malnutrition, and micronutrient deficiencies.^2,3^ Altered gut-liver axis signaling, increased intestinal permeability, and small intestinal bacterial overgrowth may further worsen hepatic injury after RYGB.^2,3^

In cases of compromised function of the gastrointestinal tract and severe malabsorption, total parenteral nutrition (TPN) is often indicated.^4^ Although long-term TPN use has been associated with parenteral nutrition–associated liver disease, recent evidence suggests that when TPN is initiated early and carefully tailored, it can serve as a therapeutic modality to reverse metabolic liver injury, especially when oral or enteral routes are not viable.^5,6^

Although there is growing recognition of nutritional support in chronic liver disease and intestinal failure, there is limited literature on the use of TPN to reverse acute liver injury, particularly in post bariatric patients. This case represents an instance of improvement of hepatic steatosis and liver injury post-RYGB through individualized TPN, highlighting the critical importance of nutritional intervention in complex postsurgical hepatic decompensation.

## CASE REPORT

A 39-year-old woman underwent sleeve gastrectomy in 2015 for obesity, followed 4 years later by RYGB due to weight regain and refractory gastroesophageal reflux. After RYGB, she experienced persistent diarrhea and gradual weight loss, which markedly worsened in the 6 months preceding admission. During this period, her weight declined from 76 kg to 58 kg—representing a 24% unintentional weight loss—and her body mass index (BMI) dropped from 32 to 25 kg/m^2^.

One month before presentation to our institution, she was evaluated for abdominal pain, nausea, vomiting, steatorrhea, and jaundice at another facility. Liver ultrasound showed hepatomegaly and features of chronic liver disease. She underwent interventional radiology trans-jugular liver biopsy with portal pressure measurements showing hepatic wedge pressure 40 mm Hg and right hepatic venous free pressure 22 mm Hg, signifying severe portal hypertension. Endoscopy (EGD) showed no varices. Liver biopsy revealed severe steatosis and moderate steatohepatitis with activity score 6/8 and fibrosis score F3.

On admission, labs were aspartate aminotransferase (AST) 66 U/L, alanine transaminase (ALT) 36 U/L, bilirubin 326 μmol/L, alkaline phosphatase 102 U/L, albumin 28 g/L, prealbumin <0.03 g/L, international normalized ratio 2.5, vitamin A 0.11 μmol/L, and vitamin D 17 nmol/L. Workup for viral hepatitis, metabolic and autoimmune liver diseases was negative; she denied alcohol intake. Imaging showed patent hepatic vasculature, mild ascites, and severe hepatic steatosis on magnetic resonance imaging. Her Model for End-Stage Liver Disease 3.0 score was 30, prompting initiation of a liver transplant evaluation.

Given her poor nutritional status, TPN was initiated 3 days post-admission after the patient refused enteral nutrition. The regimen was customized to meet protein, caloric, and micronutrient needs. She was discharged on home TPN under supervision of a multidisciplinary team including nutrition, hepatology, gastroenterology, and endocrinology.

**Figure 1.**
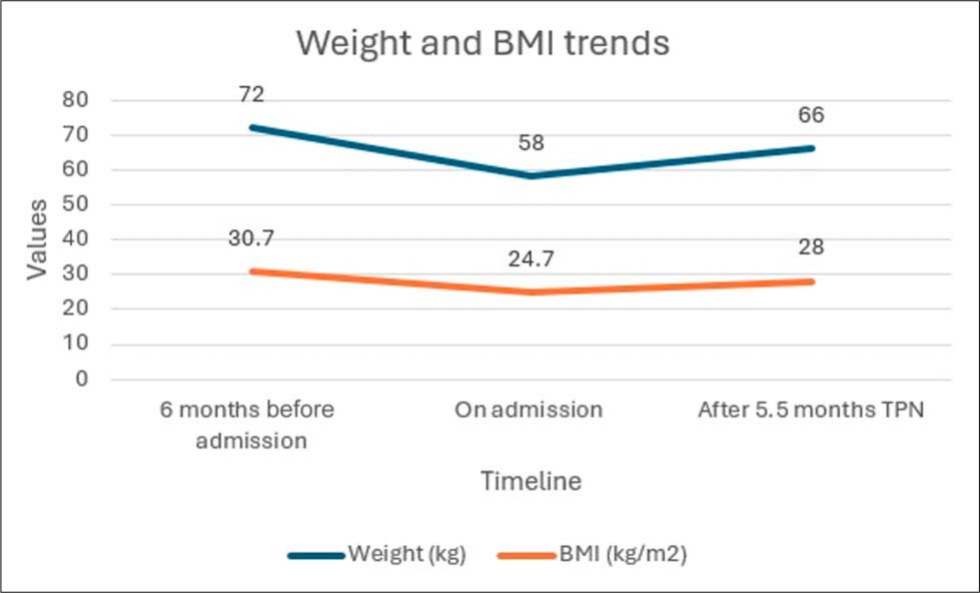
Changes in weight and BMI measured 6 months before admission, upon admission, and after 5.5 months of TPN. BMI, body mass index; TPN, total parenteral nutrition.

After 5.5 months on TPN, she gained 8 kg (66 kg, BMI 28 kg/m^2^) (Figure 1) with significant lab improvement (Table 1): AST 27 U/L, ALT 11 U/L, bilirubin 8 μmol/L, alkaline phosphatase 76 U/L, albumin 30 g/L, prealbumin 0.11 g/L, international normalized ratio 1.2, vitamin A 0.66 μmol/L, and vitamin D 110 nmol/L. Gastrointestinal symptoms resolved and she was subsequently delisted from transplant candidacy and successfully weaned off TPN.

**Table 1.** Biochemical parameters measured upon admission and after 5.5 months of TPN

	AST (U/L)	ALT (U/L)	Bilirubin (μmol/L)	Albumin (g/L)	Prealbumin (g/L)	INR	Vitamin A (μmol/L)	Vitamin D (μmol/L)
Admission	66	36	326	28	0.03	2.5	0.11	17
After 5.5 months of TPN	27	11	8	30	0.11	1.2	0.66	110

ALT, alanine transaminase; AST, aspartate aminotransferase; INR, international normalized ratio; TPN, total parenteral nutrition.

Six months after initial presentation, she underwent reversal of RYGB. Post-operatively, she maintained a stable weight, with no further hepatic decompensations.

## DISCUSSION

Acute liver injury is an uncommon but severe complication after bariatric surgery, especially in malabsorption and rapid weight loss.^2,3^ Although RYGB often improves MASLD, paradoxical hepatic decompensation can occur when nutrition is compromised.^2,7^ Early recognition and correction of malnutrition and micronutrient deficiencies are vital to prevent acute liver injury progression.^8^

This case highlights the reversibility of acute liver injury secondary to malnutrition post-RYGB through timely and sustained nutritional support with TPN. The patient achieved full recovery with 5.5 months of TPN, which corrected macro- and micronutrient deficiencies, normalized hepatic synthetic function, and led to fibrosis resolution—a rarely reported outcome. Although the operative anatomy and bowel length were unavailable, short bowel–like physiology is suspected as a contributing mechanism, consistent with reports linking excessive bypassed limb length, bacterial overgrowth, or altered nutrient transit to severe malabsorption and hepatic dysfunction even without true short bowel syndrome.

Contrary to fatal outcomes reported by Önem et al, where severe malnutrition postbariatric surgery led to death despite reversal, our patient improved with comprehensive nutritional support.^9^ Similar to findings by Lammers et al, our report underscores malnutrition as a critical driver of hepatic decompensation.^10^ Recovery in this case is likely multifactorial and mediated through correction of severe malnutrition. Sustained TPN bypassing the malabsorptive gut ensures adequate amino acid delivery and correction of fat-soluble vitamin deficiencies, thereby restoring hepatocellular protein synthesis, reducing oxidative stress, and promoting hepatocyte regeneration. In parallel, empiric pancreatic enzyme replacement therapy supported the digestive capacity in the post-RYGB setting, collectively enabling nutritional rehabilitation and hepatic recovery.^11,12^

Although evidence on TPN-mediated reversal of hepatic steatosis post-RYGB is limited, prior studies support its role in reversing nutritional liver injury. Personalized nutrition therapy has been shown to improve outcomes in liver dysfunction, and both European Society for Clinical Nutrition and Metabolism and European Association for the Study of the Liver guidelines endorse TPN when enteral feeding is not feasible.^13–15^ Van Golen et al similarly reported acute liver failure cases post-bariatric surgery where outcomes depended on early intervention.^5^

Overall, this case emphasizes that early, individualized, and multidisciplinary nutritional management—particularly with TPN—can reverse hepatic dysfunction secondary to post-bariatric malnutrition, potentially obviating the need for transplantation.

Further research is warranted to define patient selection, optimal timing, and long-term outcomes of TPN in this high-risk group.

## DISCLOSURES

Author contributions: C. Daou: Conceptualization of the case report, literature review, drafting of the manuscript, and final revision. F. Mahmoud: Literature review support and contribution to writing and editing the manuscript. D. Alkhader: Contribution to manuscript writing, formatting, and editing. A. Shatnawei: Critical revision of the manuscript for important intellectual content. SA Raheem: Critical review of the manuscript and approval of the final version. C. Daou is the article guarantor.

Financial disclosure: None to report.

Previous presentation: A poster on this case report was presented at the ESPEN Congress in Prague on September 13th.

Informed consent was obtained for this case report.

## ABBREVIATIONS:

ALT: Alanine Transaminase
AST: Aspartate Aminotransferase
BMI: Body Mass Index
EGD: Endoscopy
F: Fibrosis
IR: Interventional Radiology
MASLD: Metabolic Dysfunction–Associated Steatotic Liver Disease
MELD: Model for End-Stage Liver Disease
MRI: Magnetic Resonance Imaging
RYGB: Roux-en-Y Gastric Bypass
TPN: Total Parenteral Nutrition
